# MagmaFlow: A desktop platform for artificial intelligence‐driven expression analysis

**DOI:** 10.1002/2211-5463.70288

**Published:** 2026-06-23

**Authors:** Carlos E. Buss, Ao Li, Eduardo H. Gilglioni, Mayank Bansal, Sumeet Pal Singh, Latifa Bakiri, Alessandra K. Cardozo, Esteban N. Gurzov

**Affiliations:** ^1^ Signal Transduction and Metabolism Laboratory Université libre de Bruxelles Belgium; ^2^ Regenerative Biology Lab, Institut de Recherche Interdisciplinaire en Biologie Humaine et Moléculaire (IRIBHM), Université libre de Bruxelles Belgium; ^3^ Department of Life Sciences, School of Natural Sciences Shiv Nadar Institution of Eminence Delhi India; ^4^ Department of Laboratory Medicine Medical University of Vienna Austria

**Keywords:** Alcohol‐associated liver disease, Artificial intelligence, Computational biology, Data visualization, Volcano plots

## Abstract

While simplistic volcano plot visualizations of multi‐omics changes can highlight the most critical genomic, transcriptomic, or proteomic features, integrative frameworks combining literature evidence, pathway associations, and functional annotation remain limited. We present MagmaFlow, a cross‐platform application offering three key capabilities: literature‐based gene scoring, interactive pathway‐to‐volcano mapping, and synchronized cross‐view updates. The literature module retrieves gene associations from PubMed via PubTator3, provides direct PubMed identifier (PMID) links, and ranks genes by context‐specific relevance. The pathway module visualizes enrichment as multi‐layer circle plots displaying cross‐pathway membership, automatically synchronized with volcano selections. Interactive features include smart label positioning, drag‐and‐drop annotation, double‐click gene targeting, and customizable styles for publication‐quality figures. Thus, MagmaFlow transforms volcano plot analysis from static display into dynamic biological interpretation. To our knowledge, this is the first tool integrating artificial intelligence‐powered literature contextualization and enrichment analysis to convert differential expression data into actionable insights.

## Graphical abstract

MagmaFlow is a free, no‐code platform for gene expression analysis. It generates interactive volcano plots, links genes to literature, pathways, and diseases, prioritizes candidates using millions of publications, identifies affected biological processes, builds network diagrams, and exports publication‐ready figures and reports for macOS and Windows.

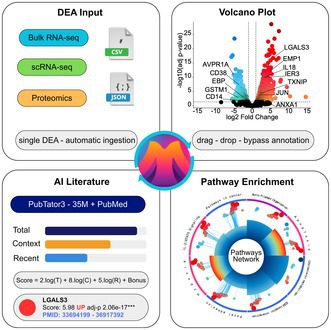

**Abbreviations**
ADH: alcohol dehydrogenase
AGE‐RAGE: advanced glycation end‐product/receptor for advanced glycation end‐products
AI: artificial intelligence
ALD: alcohol‐associated liver disease
API: application programming interface
CSV: comma‐separated values
DEA: differential expression analysis
ECM: extracellular matrix
EMT: epithelial–mesenchymal transition
FDR: false discovery rate
GB: gigabyte
GEO: gene expression omnibus
GO: gene ontology
GPU: graphics processing unit
GSEA: gene set enrichment analysis
GUI: graphical user interface
HD: high definition
IGF: insulin‐like growth factor
IL: interleukin
JDK: Java Development Kit
JSON: JavaScript Object Notation
KEGG: Kyoto Encyclopedia of Genes and Genomes
LTS: long‐term support
MeSH: Medical Subject Headings
MSigDB: molecular signatures database
NCBI: National Center for Biotechnology Information
NF‐κB: nuclear factor kappa B
PMC: PubMed Central
PMID: PubMed identifier
PNG: Portable Network Graphics
PPAR: peroxisome proliferator‐activated receptor
PPI: protein–protein interaction
RAM: random access memory
RNA‐seq: RNA sequencing
RUVSeq: remove unwanted variation sequencing
scRNA‐seq: single‐cell RNA sequencing
SOCS: suppressor of cytokine signaling
STRING: Search Tool for the Retrieval of Interacting Genes/proteins
TNF: tumor necrosis factor
URL: uniform resource locator
UTF‐8: 8‐bit Unicode Transformation Format
UV: ultraviolet

High‐throughput technologies have grown exponentially over the past decade, enabling important findings across diverse omics platforms [1]. Platforms such as Illumina, 10× Genomics, MGI Tech, Oxford Nanopore, and PacBio are producing high‐resolution molecular data from single cells to whole tissues [2, 3]. The four core fields of genomics, transcriptomics, proteomics, and metabolomics have expanded to include epigenomic technologies, which capture heritable regulatory modifications beyond DNA sequence, six interactomics categories encompassing protein–protein, gene–protein, protein–metabolite, protein–DNA, protein–RNA, and chemical–protein interaction networks, and spatial omics approaches that preserve tissue context [1]. Together, these advances have created a critical demand for powerful analysis tools capable of handling increasingly diverse and complex datasets while ensuring quality control and publication‐ready outputs [4].

Volcano plots remain a central resource for differential expression analysis, combining effect size and statistical significance to help researchers prioritize genes of interest for downstream experimental validation [5]. However, even after significance filtering, datasets frequently retain hundreds or thousands of differentially expressed features, resulting in crowded visualizations where only the most extreme changes receive attention while biologically relevant genes in intermediate positions remain overlooked [5]. For wet‐laboratory researchers designing follow‐up experiments, identifying the right candidates from this abundance of information is a critical and often time‐consuming bottleneck.

This challenge extends beyond visualization, reflecting a broader difficulty in interpreting multi‐omics data, that is, making biological sense of measurements collected simultaneously across multiple molecular platforms. Annotating differentially expressed genes requires systematic integration of molecular function signatures, pathway‐level over‐representation analysis, gene network relationships, and disease associations across several databases [6]. Although manual annotation can be accurate, it becomes increasingly impractical and time‐consuming given the volume and complexity of high‐throughput datasets [6, 7].

Current volcano plot tools perform well for basic visualization but are not equipped with comprehensive gene annotation at their core [5]. Programming solutions such as ggplot2 (https://ggplot2.tidyverse.org) and Matplotlib (https://matplotlib.org) offer reproducible, high‐quality outputs but require coding expertise [5, 8], while graphical alternatives partially improve accessibility at the cost of interactivity and scalability [8, 9]. Features such as automatic repositioning of gene labels to prevent overlapping text in dense figures, and intelligent routing of connector lines between labels and data points, remain largely unavailable in both approaches, leaving researchers to choose between publishing unlabeled volcano plots or figures crowded with overlapping annotations [5].

In addition, most existing tools lack integration with biomedical literature and pathway enrichment resources [5]. Gene‐specific information must be retrieved manually from external databases, and pathway enrichment analysis remains an isolated analytical step, with results presented separately rather than mapped back to the expression data [10]. This fragmented workflow imposes a significant burden, particularly for teams without dedicated bioinformatics support [10, 11], highlighting the need for unified solutions that bridge visualization, annotation, and biological contextualization in a single environment [10].

To address these challenges, we developed MagmaFlow, a standalone cross‐platform application combining interactive volcano plot visualization with automated annotation, integrated literature mining, and pathway‐level contextual analysis. MagmaFlow retrieves relevant PubMed references, pathway memberships, and disease associations directly within an interactive visualization environment, enabling researchers to move from raw differential expression results to biologically contextualized candidate genes, proteins, or metabolites without switching between tools or requiring programming skills. By streamlining these functions in a single platform, MagmaFlow enables efficient, reproducible, and publication‐ready analysis of differential expression datasets across transcriptomic, proteomic, and single‐cell platforms. We evaluated MagmaFlow using differential expression datasets from alcohol‐associated liver disease (ALD) as a test case, with an extended multi‐omics integration analysis. ALD pathogenesis involves concurrent dysregulation across metabolic, inflammatory, and fibrotic axes, where key regulators participate in multiple pathways simultaneously, with stress response transcription factors connecting to inflammatory cascades while lipid metabolism genes intersect with fibrogenic signaling [12, 13]. This setting is well‐suited for evaluating MagmaFlow's candidate gene prioritization and cross‐pathway visualization capabilities, while also serving as a reminder that highly connected genes often reflect generic responses, such as oxidative stress and inflammation, that are common to many diseases and should therefore be interpreted with contextual evidence.

## Materials and methods

### Software availability

MagmaFlow is freely available for academic and noncommercial use under an End‐User License Agreement from Université libre de Bruxelles (ULB). The source code is maintained as closed‐source under the stewardship of the knowledge transfer office (KTO)‐ULB, which holds the intellectual property rights to the Software. Cross‐platform binaries for macOS and Windows, complete documentation, and example datasets are available at https://doi.org/10.5281/zenodo.20299121 and https://github.com/carlosbuss1/MagmaFlow. Minimum system requirements are 4 gigabytes (GB) random access memory (RAM) and a 1280 × 720 pixel display. For large datasets (> 30 000 genes), 8 GB RAM and a full high definition (HD) display are recommended. macOS users may encounter a Gatekeeper security prompt upon first launch; the MagmaFlow binary is code‐signed and notarized through the Apple Developer Program and detailed installation instructions are provided on the MagmaFlow website and in the bundled README file.

### Technical implementation

The application was developed using JavaFX 17.0.2 and compiled with Java Development Kit (JDK) 17 long‐term support (LTS), following Model‐View‐Controller architecture. Rendering utilizes the JavaFX Canvas API with graphics processing unit (GPU)‐accelerated GraphicsContext for 2D vector graphics https://openjfx.io/. The plotting engine employs bidirectional coordinate transformation, converting gene coordinates in log_2_ fold change and negative log_10_ *P*‐value space to canvas pixel coordinates via linear interpolation with dynamic axis scaling. Logarithmic transformations are constrained by minimum thresholds to prevent undefined values, and all computations use double‐precision floating‐point arithmetic.

### Input requirements and upstream data processing

**Fig. 1:**
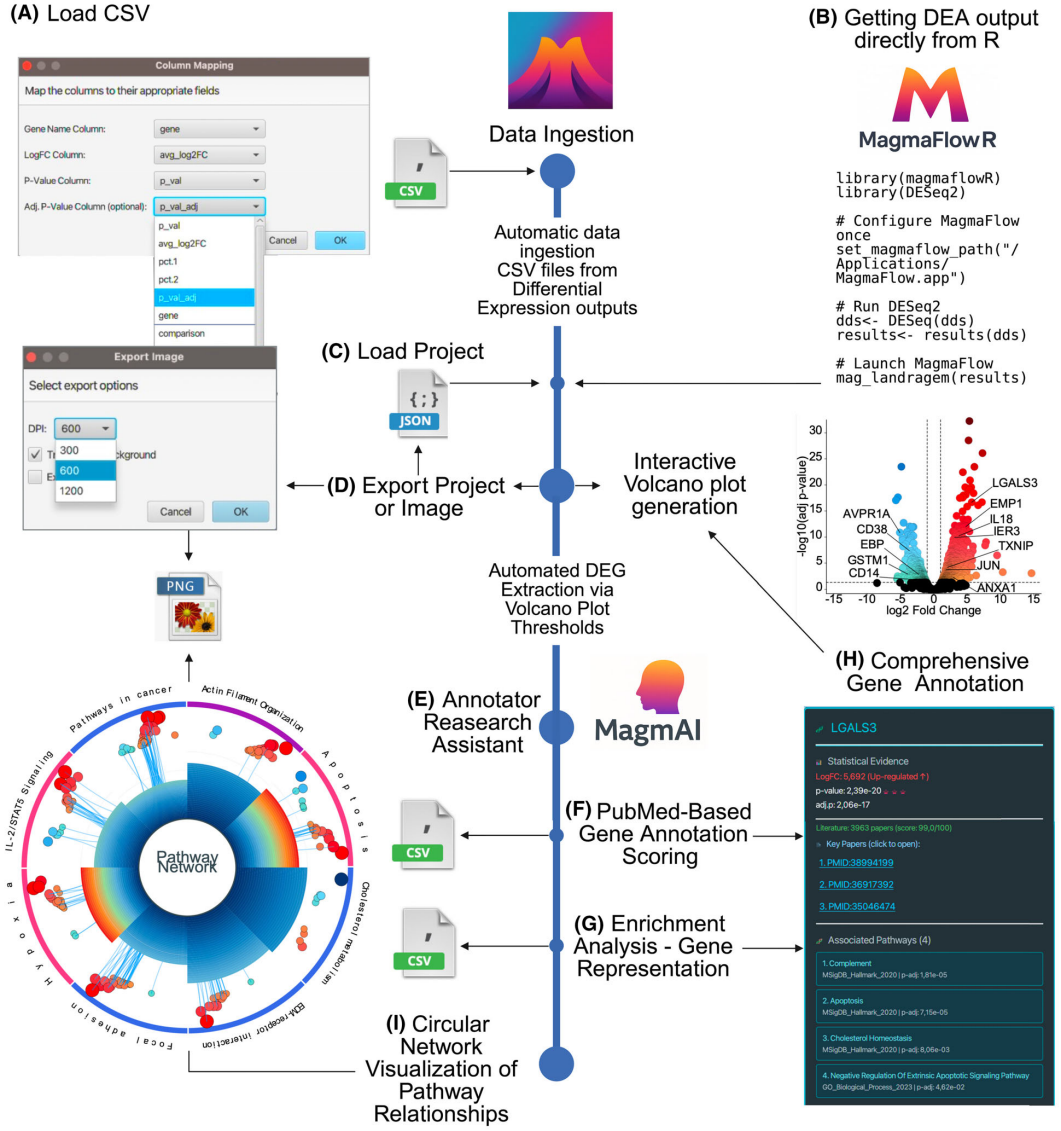
MagmaFlow Software Architecture and Workflow. The platform integrates data processing, visualization, and AI‐assisted annotation through a modular architecture. (A) Data import pathway for CSV files derived from standard differential expression analysis tools (DESeq2, edgeR, limma, Seurat), applying a dynamic column‐mapping interface for automatic or manual detection of key statistical parameters. (B) Direct integration via the MagmaFlowR plugin allows real‐time synchronization within the R environment. (C) Project state management enables the loading of preconfigured JSON files to fully restore visualization parameters and layouts. (D) High‐resolution export options support PNG generation (300–1200 dpi) and complete project preservation via JSON. (E) The MagmAI module serves as an integrated research assistant, generating biological annotations through (F) the gene scoring system utilizing the PubTator3 API/PubMed data and (G) enrichment analysis using the EnrichR API. (H) Comprehensive labels derived from PubMed scoring and predicted biological processes. (I) An interactive Pathway Analysis feature facilitates the selection of key pathways to generate Circular Network visualizations revealing pathway relationships. Figure created in BioRender: Gurzov, E. (2026) https://BioRender.com/jzu6edc.

MagmaFlow accepts as direct input the tabular output of differential expression analysis in comma‐separated values (CSV) format, containing gene or protein identifiers, log_2_ fold change values, *P*‐values, and adjusted *P*‐values, and is compatible with results from bulk RNA sequencing (RNA‐seq), single‐cell RNA‐seq (scRNA‐seq), proteomics, phosphoproteomics, and other quantitative omics platforms. The platform processes a single differential expression analysis (DEA) result at a time and does not perform normalization or batch effect correction internally; these steps are expected to be applied upstream using tools appropriate to the platform and experimental design. Upon import, a default significance filter of adjusted *P*‐value ≤ 0.05 and |log_2_ fold change| ≥ 1 defines the active gene list used as the basis for all downstream analyses, with both thresholds fully customizable. As illustrated in Fig. 1, the interactive volcano plot serves as the analytical hub, simultaneously distributing the filtered gene list to the literature annotation and enrichment modules while receiving and integrating results returned by the external APIs.

### External data resources and application programming interfaces

MagmaFlow integrates three external data resources accessed dynamically at runtime via their respective public APIs, subject to each provider's terms of service. No data are stored locally within the application; all queries are performed on demand during an active user session.

Literature mining is powered by the PubTator3 API (https://www.ncbi.nlm.nih.gov/research/pubtator3/) [14], which provides artificial intelligence (AI)‐powered named entity recognition across approximately 36 million PubMed abstracts and 6 million PubMed Central (PMC) full‐text articles. Gene‐to‐publication co‐occurrence data are retrieved via the /relations endpoint, returning total publication counts, disease‐contextual publication counts, and supporting evidence snippets for each queried gene. Date‐filtered recent publication counts and PubMed identifiers are retrieved independently via National Center for Biotechnology Information (NCBI) E‐utilities (https://www.ncbi.nlm.nih.gov/books/NBK25501/) [15], using the ESearch endpoint with a default date window from 2020 onwards, customizable by the user. Pathway enrichment analysis is performed via the Enrichr API (https://maayanlab.cloud/Enrichr/) [16, 17] using Over‐Representation Analysis based on Fisher's exact test with Benjamini–Hochberg false discovery rate (FDR) correction. The following database releases are queried: Gene Ontology 2026 (Biological Process, Molecular Function, and Cellular Component) [18, 19], Kyoto Encyclopedia of Genes and Genomes (KEGG) 2026 [20], Reactome 2024 [21], WikiPathways 2024 [22], and Molecular Signatures Database (MSigDB) Hallmark 2025 [23]. Database release versions are explicitly specified in each API call to ensure reproducibility of enrichment results across MagmaFlow versions.

All input datasets, intermediate outputs, and enrichment results used to generate the figures presented in this manuscript are publicly available in the MagmaFlow GitHub repository (https://github.com/carlosbuss1/MagmaFlow), enabling full independent reproduction of all validation analyses without requiring access to the MagmaFlow binary.

### Literature mining and gene relevance scoring

For each significant gene, three complementary literature metrics are retrieved via a dual‐API strategy. The total publication count is obtained from the PubTator3/relations endpoint using a general gene‐entity association query. The contextual publication count is obtained from the same endpoint with an additional disease‐entity constraint, reflecting publications co‐annotating the gene and the user‐specified disease context. The recent publication count is retrieved via NCBI ESearch [15] filtered to human studies published from 2020 onwards by default, with the date window fully customizable by the user. These metrics are combined into a composite relevance score:

$$\text{Score}=\begin{array}{l} 2\cdot \log\left(1+\text{Total}\right)+8\cdot \log\left(1+\text{Context}\right)\\ +\;5\cdot \log\left(1+\text{Recent}\right)+\text{StatsBonus} \end{array}$$

Logarithmic scaling prevents highly studied genes from dominating the ranking. The contextual term carries the highest weight (coefficient 8) to prioritize disease relevance. A statistical bonus of up to 10 points is added based on the gene's adjusted *P*‐value (up to 5 points) and absolute log_2_ fold change (up to 5 points). This scoring reflects literature co‐occurrence frequency and statistical salience rather than functional or causal inference and should be interpreted as a hypothesis‐prioritization metric. The system operates in two phases: Phase 1 retrieves literature counts for all significant genes (three API calls per gene); Phase 2 retrieves up to five PMIDs for the top 50 ranked genes using disease synonym expansion to maximize retrieval specificity. Genes with zero total publications are excluded from output.

### Pathway enrichment module: API Integration

Pathway enrichment analysis is implemented in the Pathway Enrichment Analyzer class, which interfaces with the Enrichr API (https://maayanlab.cloud/Enrichr) via two sequential HTTP requests. In the first request, the filtered gene list is submitted to the *addList* endpoint as a multipart/form‐data POST request with a dynamically generated boundary string. The response returns a *userListId* that uniquely identifies the submitted gene set on the Enrichr server.

In the second request, enrichment results are retrieved for each selected database library by querying the enrich endpoint with the userListId and the library name as uniform resource locator (URL) parameters. Responses are returned as JSON objects keyed by library name, containing arrays of pathway results in the 2024 Enrichr API format: rank, term name, *P*‐value, odds ratio, combined score, gene array and adjusted *P*‐value. Results are filtered at adjusted *P*‐value < 0.05 and sorted by adjusted *P*‐value, retaining the top 50 enriched terms per library.

Pathway term sizes are retrieved from the Enrichr *geneSetLibrary* endpoint prior to enrichment querying, loading the complete gene set definitions for each library and caching pathway sizes in memory. These sizes are used to construct gene set overlap strings in the format *overlapSize/termSize* and to ensure accurate representation of enrichment statistics. For Gene Ontology (GO) terms, pathway identifiers are extracted from parenthetical GO accession numbers; for WikiPathways terms, identifiers are extracted from the WP‐prefixed suffix, and for KEGG and Reactome terms, species suffixes are removed to generate clean pathway names.

### Circle plot rendering

Circle plots are rendered using the JavaFX Canvas API. Pathways are arranged radially around a central hub, with angular sectors allocated proportionally to the number of member genes. Individual genes are positioned within their respective pathway sectors at a radial distance proportional to their negative log_10_ adjusted *P*‐value and at an angular position within the sector proportional to their log_2_ fold change rank. Genes appearing in multiple pathways are connected by curved Bezier lines to indicate shared membership across pathway sectors. Bar heights at each pathway arc represent odds ratios, with gradient coloring encoding adjusted *P*‐values. A dedicated customization console allows full configuration of plot dimensions, node sizes, label font sizes, label orientation, edge weights and color schemes.

### Gene‐pathway network analysis

To further explore cross‐pathway relationships, gene‐pathway network analysis was performed entirely outside MagmaFlow, using MagmaFlow's circle plot enrichment results as input to a dedicated Python‐based network analysis workflow. Network nodes represent genes, with node size scaled proportionally to degree centrality, reflecting the number of pathway connections per gene. Edges connect gene pairs participating in at least one shared pathway, with intra‐cluster edges rendered as straight lines and inter‐cluster edges as curved lines to distinguish local pathway relationships from cross‐tier functional connections. Network statistics including node count, edge count, and top hub genes ranked by degree centrality were calculated using the Python NetworkX library. This analysis was performed to illustrate the downstream applicability of MagmaFlow enrichment outputs and does not reflect a native capability of the platform.

### Multi‐omics integration analysis

To illustrate the compatibility of MagmaFlow outputs with broader multi‐omics study designs, we performed an integrative analysis using publicly available alcoholic liver disease (ALD) datasets spanning transcriptomic and proteomic platforms. This analysis was performed entirely outside MagmaFlow, using its differential expression outputs as inputs to dedicated external integration workflows. MagmaFlow is an essentially univariate tool that processes a single DEA result at a time and is not designed as a multi‐omics integration platform.

Bulk RNA‐seq data were obtained from Gene Expression Omnibus (GEO) accession GSE142530. Sample‐level variability was assessed by principal component analysis and remove unwanted variation sequencing (RUVSeq) was applied to estimate and remove unwanted variation, with the resulting correction factors included as covariates in the differential expression model. Single‐cell RNA‐seq data were obtained from GSE255772. Seurat's CCA‐based integration was used to handle batch effects across samples for cell cluster identification and annotation, after which pseudo‐bulk differential expression analysis was performed on the annotated clusters [24]. For proteomic and phosphoproteomic data, processed differential expression results were used directly as reported by Hardesty et al. [25] (MassIVE: MSV000089168), where normalization and statistical modeling had been performed according to the original study's analytical framework. Differential expression criteria of *P*‐value < 0.05 and |log_2_ fold change| > 1 were applied consistently across all transcriptomic datasets.

Each dataset was independently analyzed in MagmaFlow to generate enrichment outputs, and cross‐platform concordance was subsequently assessed by identifying overlapping differentially expressed genes and proteins across datasets. Consensus pathways appearing in two or more platforms were identified and ranked using a composite scoring scheme incorporating platform validation (0–30 points), statistical significance (0–40 points), and gene multi‐platform coverage (0–30 points). Nonliver‐specific pathways were excluded, and the top 10 ALD‐relevant pathways were selected for visualization. Venn diagrams were generated using matplotlib‐venn (Python), integrated heatmaps using ComplexHeatmap [26], and Sankey diagrams as interactive React components.

### Comparative feature analysis

Fourteen volcano plot visualization tools were systematically evaluated across 31 features in four categories: interactive visualization, usability and accessibility, AI‐powered literature integration, and pathway enrichment analysis. Feature support was classified as full, partial, or absent. Results were visualized using a modified UpSet plot [27] (Python/Matplotlib) with tools sorted by feature count, gradient‐colored matrix circles indicating support level, and marginal bar charts displaying feature distributions.

### Statistical methods

Pathway enrichment significance is assessed using Fisher's exact test with Benjamini‐Hochberg FDR correction, with pathways considered significantly enriched at adjusted *P*‐value < 0.05.

## Results

### Software architecture and user interface

MagmaFlow is deployed as a standalone executable enabling zero‐configuration installation. The user interface comprises three SplitPane panels: the left panel provides volcano plot settings including threshold adjustments, color mapping, and gene filtering; the central canvas displays the interactive volcano plot visualization; and the right panel houses the MagmAI Research Assistant featuring PubMed‐based gene annotation via AI‐powered PubTator3 API [14], pathway enrichment analysis via Enrichr integration [16, 17, 28], and circle plot configuration for pathway network visualization (Fig. 1).

DEA results from diverse multi‐omics layers (transcriptomics, proteomics, metabolomics, etc.) are supported, independent of their analytical origin (R https://www.R‐project.org/, Python https://www.python.org/, or other platforms). Datasets are imported in CSV format, with automatic detection of standard columns (gene/feature ID, log_2_ fold change, *P*‐value, adjusted *P*‐value) via regular expressions; nonstandard headers can be manually assigned through an interactive mapping dialog. 8‐Bit Unicode Transformation Format (UTF‐8) encoding is enforced, and the system handles common delimiter types and decimal notations automatically. Following established patterns for high‐performance genomic visualization tools, gene data are stored as objects with numerical and label attributes, while annotation states and coordinates are managed using hash maps for efficient rendering and interaction (Fig. 1A). The MagmaFlowR companion package provides integration with R‐based differential expression pipelines, including DESeq2 [29], edgeR [30], limma [31], and Seurat [24]. The *mag_landragem*() function launches the MagmaFlow graphical user interface (GUI) and preloads expression data directly from the R environment (Fig. 1B). Project files are saved in JavaScript Object Notation (JSON) format, preserving gene data, statistical thresholds, annotations, label positions, and display preferences (Fig. 1C). The application automatically tracks unsaved changes to prevent data loss. High‐resolution Portable Network Graphics (PNG) images can be exported using scalable off‐screen rendering (Fig. 1D).

The MagmAI module operates as an integrated research assistant (Fig. 1E), implementing a gene scoring system that queries the PubTator3 API [14] to retrieve PubMed‐indexed publications and calculates relevance scores based on literature frequency and research context (Fig. 1F). Pathway enrichment analysis is performed through automated EnrichR API [16, 17] queries, identifying significantly enriched biological processes, molecular functions, and cellular components from multiple ontology databases (Fig. 1G). Comprehensive gene annotations are generated by combining PubMed‐derived scoring metrics with predicted biological processes, enabling rapid identification of functionally relevant differentially expressed genes (Fig. 1H). The interactive pathway analysis module allows users to select key enriched pathways and generates circular network visualizations that reveal functional relationships and pathway crosstalk patterns (Fig. 1I).

### Interactive visualization and dynamic label management

**Fig. 2:**
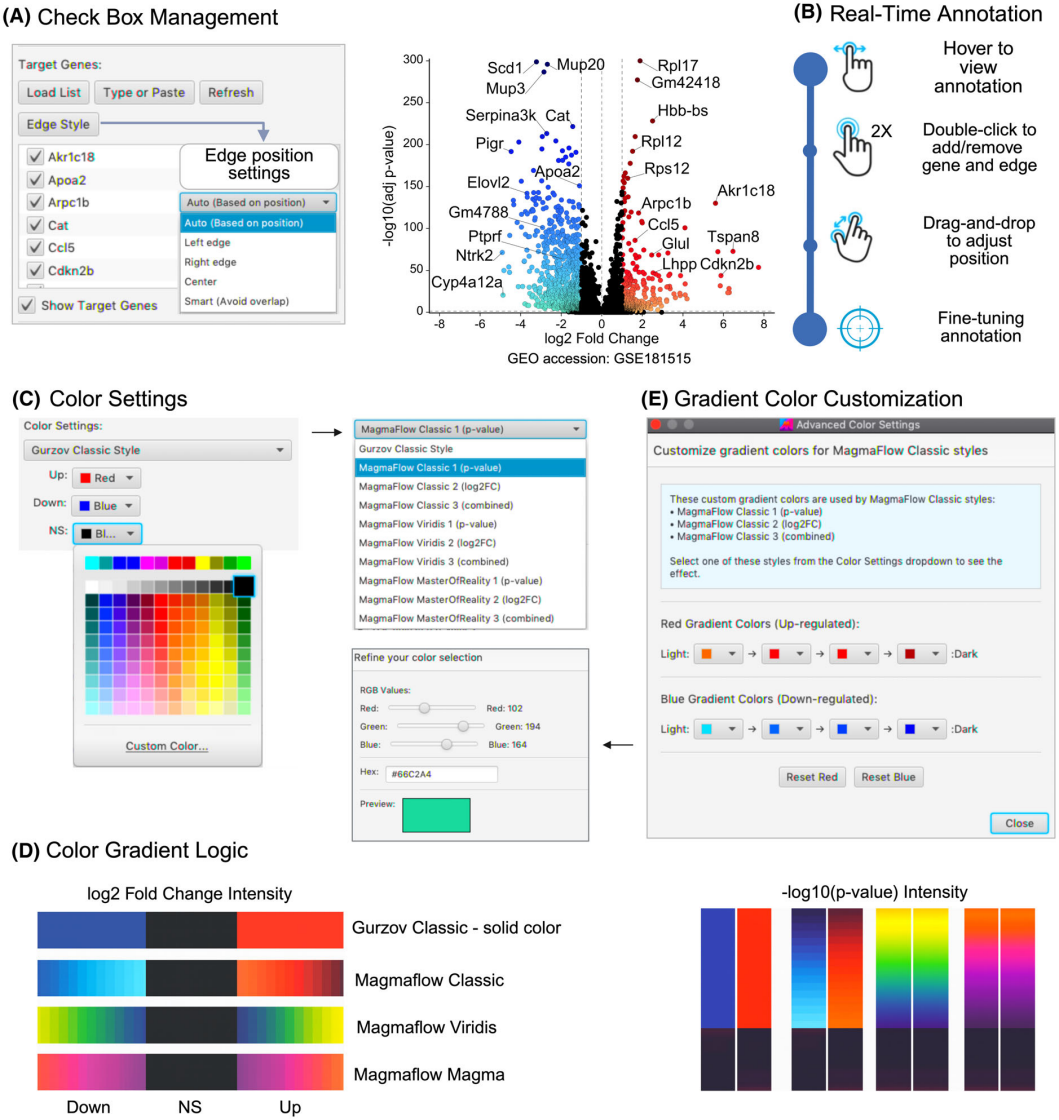
Interactive annotation, label management, and color customization in MagmaFlow. (A) Users can manage target gene labels through synchronized checkboxes in the target panel, allowing real‐time activation or deactivation of annotations on the volcano plot. (B) Interactive exploration features include hover tooltips displaying the gene name, log_2_ fold change, *P*‐value, and adjusted *P*‐value. Double‐clicking any data point adds or removes it from the target list. Labels can be freely repositioned via drag‐and‐drop, allowing for manual placement and improved readability, and edge placement is optimized through five layout modes (Auto, Left, Right, Center, and Smart) to reduce overlap. (C) MagmaFlow offers flexible color mapping, including a customizable style solid palette (red for up‐regulated, blue for downregulated, and black for nonsignificant genes) and (D) three gradient modes: Classic (light‐to‐dark blue for downregulated, orange‐to‐dark red for up‐regulated), and color‐blind‐friendly palettes Viridis and Magma. (E) Fine‐tuning of gradient color customization. Figure created in BioRender. Gurzov, E. (2026) https://BioRender.com/7fh794g.

Threshold lines for fold change and statistical significance are rendered dynamically and updated in real time. Target gene annotations are managed through synchronized checkboxes in the target panel, enabling real‐time activation or deactivation of individual gene labels directly on the volcano plot (Fig. 2A). Interactive exploration features include hover tooltips displaying gene name, log_2_ fold change, *P*‐value, and adjusted *P*‐value for rapid data inspection. Double‐clicking any data point adds or removes it from the target list, while labeled genes can be freely repositioned via drag‐and‐drop for manual placement optimization. Edge placement is further refined through five automated layout modes (Auto, Left, Right, Center, and Smart) that minimize label overlap and maximize readability (Fig. 2B).

The application supports binary and gradient‐based color schemes mapping to log_2_ fold change, *P*‐values, or combined metrics. Perceptually uniform color palettes include adjustable outlines and transparency to reduce overplotting. Binary coloring uses a customizable solid palette for up‐regulated (red), downregulated (blue), and nonsignificant genes (black) (Fig. 2C), while gradient modes include Classic (light‐to‐dark blue for downregulated, orange‐to‐dark red for up‐regulated), Viridis, and Magma palettes (Fig. 2D), with fine‐tuning controls for color transitions and intensity adjustments (Fig. 2E).

### AI‐powered literature mining module

To automatically rank differentially expressed genes based on literature evidence aligned with the user's research context, MagmaFlow integrates the PubTator3 API and NCBI E‐utilities in a streamlined dual‐API strategy, leveraging AI‐powered named entity recognition across over one billion annotations spanning 36 million PubMed abstracts and 6 million PMC full‐text articles [14].

**Fig. 3:**
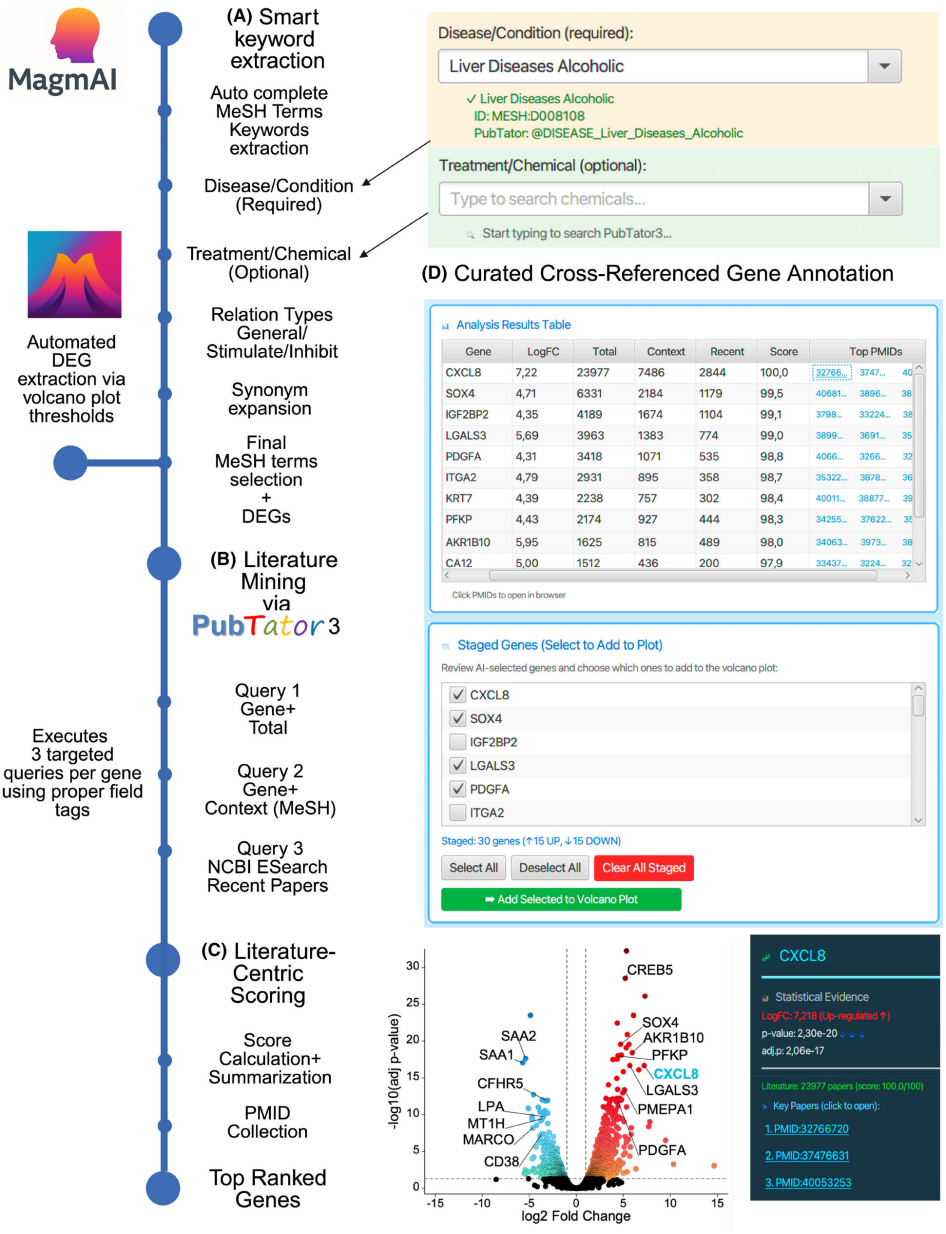
Literature Mining module integrating PubTator3 semantic entity resolution with NCBI E‐utilities. (A) Context Definition: Users define research context through PubTator3 dynamic autocomplete, selecting a Disease or Condition (required) and optional Treatment or Chemical, with validated MeSH identifiers. Relation Types can be configured to match expected expression patterns including positive or negative correlation, stimulation, inhibition, or general association. (B) Literature Mining: For each significant gene, three complementary metrics are retrieved through a dual‐API strategy. Total Papers and Context‐Relevant Papers are queried via the PubTator3 Relations API, returning publication counts for gene associations and gene‐disease relationship pairs. Recent Papers are retrieved via NCBI E‐utilities with data filtering defaulting to 2020 onwards and fully customizable by the user. (C) Literature‐Centric Scoring: A log‐scaled composite score weights context‐relevant papers most heavily while preventing highly studied genes from dominating results. PMID retrieval for the top 50 ranked genes employs disease synonym expansion to maximize specificity. (D) Curated Cross‐Referenced Gene Annotation: Users can review ranked genes with evidence summaries and clickable PMID links sorted by publication date, select key targets, and integrate annotations directly into the volcano plot with literature‐enhanced tooltips. Figure created in BioRender. Gurzov, E. (2026) https://BioRender.com/a5qaond.

Context definition employs dynamic entity autocomplete through the PubTator3 API, with users selecting a disease or condition as the required context and an optional treatment or chemical specification, receiving validated entity suggestions with standardized Medical Subject Headings (MeSH) identifiers [14] (Fig. 3A). The system additionally allows selection of PubTator3 relation types to refine the biological context, including positive or negative correlation between genes and diseases, stimulation or inhibition of genes by chemicals, and general association, enabling users to align literature queries with the expected expression patterns of their differential analysis.

Three complementary literature metrics are retrieved for each significant gene and combined into a composite relevance score as described in the Methods (Fig. 3B). Genes are ranked by this score, and the top candidates are returned with evidence summaries, clickable PMID links sorted by publication date, and volcano plot integration (Fig. 3C,D). Output is available as CSV export for downstream use.

### Pathway enrichment and circle plot visualization

**Fig. 4:**
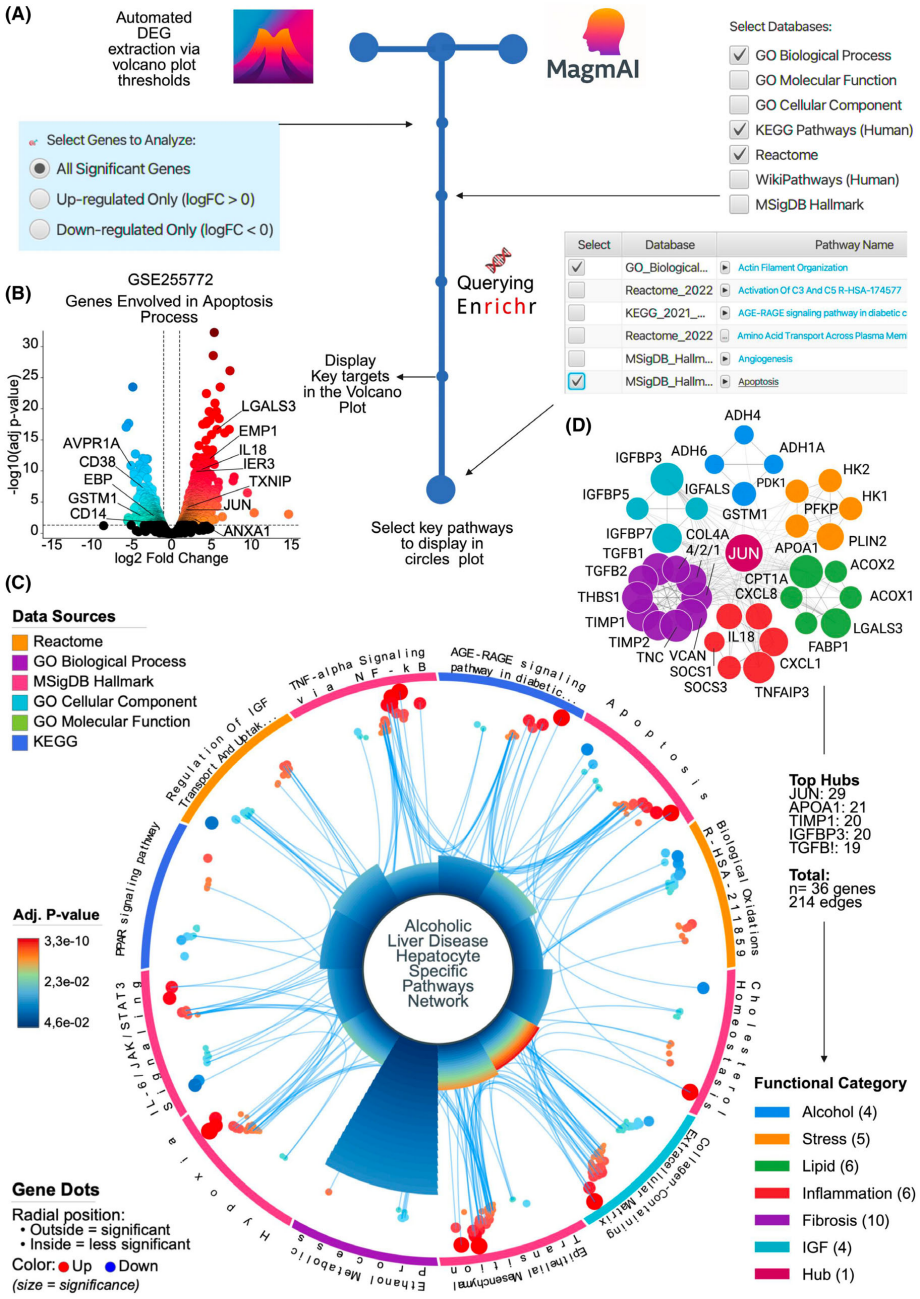
Integrated pathway enrichment analysis with interactive visualization in MagmaFlow. (A) EnrichR API Integration: MagmaFlow submits differentially expressed genes to the EnrichR web service, querying multiple pathway databases (GO Biological Process, GO Molecular Function, KEGG, Reactome, WikiPathways, and MSigDB Hallmark) simultaneously. Results include *P*‐values, adjusted *P*‐values, odds ratios, and combined scores for rapid functional characterization. (B) Volcano Plot Pathway Annotation: Enriched pathway gene sets are dynamically linked to the volcano plot. Upon pathway selection, member genes are automatically highlighted and labeled, enabling assessment of statistical significance and fold change distribution within specific biological pathways. (C) Pathway Network Circle Plot: Selected pathways are visualized as a multi‐layered circular diagram displaying pathway enrichment significance (inner ring), gene expression levels (middle ring), and database source annotations (outer ring), facilitating identification of pathway interconnections and prioritization of regulatory modules for experimental validation. (D) ALD Mechanistic Gene Network: To further contextualize the enrichment results obtained from MagmaFlow, a downstream network visualization was constructed independently using R (igraph package). Genes from twelve selected pathways are organized into six functional clusters based on shared pathway membership: Alcohol Metabolism (Ethanol Metabolic Process, Biological Oxidations), Stress Response (Hypoxia, Apoptosis), Lipid Metabolism (Cholesterol Homeostasis, PPAR Signaling), Inflammatory Signaling (TNF‐α/NF‐κB, IL‐6/JAK/STAT3), Fibrosis/ECM (EMT, Collagen‐Containing ECM), and Metabolic Complications (AGE‐RAGE Signaling, IGF Transport/IGFBPs). Node size reflects connectivity (number of shared pathways), node color indicates functional category, and edges represent comembership in at least one pathway. This network visualization reveals hub genes such as JUN connecting multiple biological processes and identifies cross‐tier relationships between distinct functional modules. Figure created in BioRender. Gurzov, E. (2026) https://BioRender.com/duqo2io.

MagmaFlow integrates pathway enrichment analysis directly within the visualization environment, identifying biological processes modulated by differentially expressed genes and enabling researchers to detect coordinated functional changes across gene sets. Three analysis modes are available: enrichment using all significant genes combined, or separate fold change‐stratified gene set enrichment analysis for upregulated and downregulated gene subsets submitted independently to the Enrichr API (Fig. 4A). In the stratified mode, MagmaFlow partitions the filtered gene list based on the sign of the log_2_ fold change and submits each subset independently, allowing users to identify biological processes statistically overrepresented among genes increasing or decreasing in abundance under the experimental condition.

MagmaFlow queries the Enrichr API (https://maayanlab.cloud/Enrichr/) [16, 17] to perform Over‐Representation Analysis across seven pathway databases: Gene Ontology 2026 (Biological Process, Molecular Function, Cellular Component) [18, 19], KEGG 2026 [20], Reactome 2024 [21], WikiPathways 2024 [22], and MSigDB Hallmark 2020 [23]. All databases are available for Human and Mouse, while Cattle (*Bos taurus*) and Zebrafish (*Danio rerio*) are limited to Gene Ontology, with partial KEGG coverage for zebrafish.

**Fig. 5:**
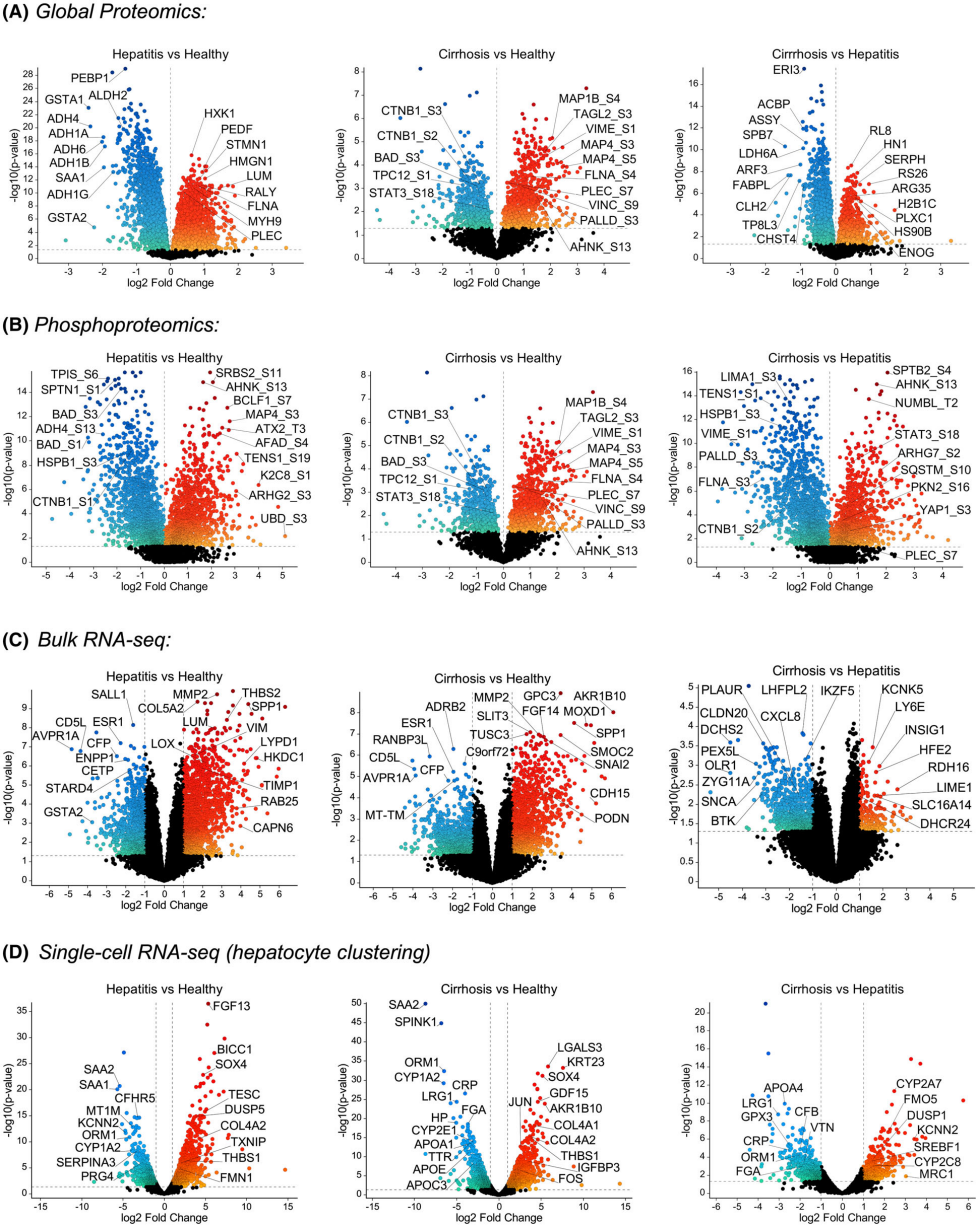
Cross‐omics differential expression analysis visualization of Alcohol‐Associated Liver Disease (ALD) transitions in MagmaFlow. (A) Global Proteomics: Volcano plots for Alcoholic Hepatitis vs Healthy, Cirrhosis vs Healthy, and Cirrhosis vs Alcoholic Hepatitis, showing global protein abundance changes across ALD progression. (B) Phosphoproteomics (pSer): Matching transitions at the phosphosite level, highlighting phosphorylation‐specific regulation. Both datasets were obtained from [25] (MassIVE: MSV000089168). (C) Bulk RNA‐seq: Matching transitions visualized from GEO: GSE142530 liver biopsies (Alcoholic Hepatitis, Cirrhosis, Healthy), processed with a standard differential expression analysis pipeline and imported into MagmaFlow. (D) Single‐cell (hepatocyte‐resolved): GEO: GSE255772 single‐cell liver data (severe alcohol‐associated hepatitis, alcohol‐associated cirrhosis, and healthy). Hepatocyte clusters were subset and contrasted in MagmaFlow to display per‐cluster differential expression analysis volcano plots for ALD transitions.

As a proof of principle, we analyzed hepatocyte‐specific datasets with MagmaFlow, comparing ALD versus healthy liver (Figs 4A–D and 5A–D), and then performed multi‐omics integration using MagmaFlow outputs (Fig. S1A–F, [25]). Genes exhibiting multi‐pathway membership are classified as high‐priority targets due to their functional connectivity across biological processes [32]. These candidates can be directly annotated on the volcano plot, enabling an integrated analysis in which pathway enrichment guides gene prioritization while volcano visualization places pathway‐associated genes within their differential expression statistics (Fig. 4B).

Results are visualized using circle plots where enriched pathways are arranged radially with individual genes represented as dots. Gene positioning reflects statistical significance via radial distance and fold change direction via angular position within pathway sectors. Dot colors indicate fold change direction, with red for upregulated and blue for downregulated genes, and intensity proportional to significance level. Pathway enrichment bars display odds ratios as bar height with gradient coloring representing adjusted *P*‐values. Curved lines connect genes appearing in multiple pathways, highlighting shared gene membership across enriched pathways and serving as a basis for hypothesis generation regarding potential pathway interactions. It should be noted that these connections reflect gene‐sharing networks rather than experimentally validated or mechanistically established pathway crosstalk and should not be interpreted as direct evidence of biological pathway interaction. Color bands identify the source database for each pathway.

### Pathway discovery in hepatocyte scRNA‐seq: A validation study

Pathway enrichment analysis identified 110 significantly enriched pathways across six databases (GO Biological Process, GO Cellular Component, GO Molecular Function, MSigDB Hallmark, Reactome, KEGG). From these, twelve pathways were selected based on significance and biological relevance to ALD pathophysiology, organized into six functional clusters: alcohol metabolism (Ethanol Metabolic Process, Biological Oxidations), stress response (Hypoxia, Apoptosis), lipid metabolism (Cholesterol Homeostasis, peroxisome proliferator‐activated receptor (PPAR) Signaling), inflammatory signaling (tumor necrosis factor (TNF)‐α/nuclear factor kappa B (NF‐κB), interleukin (IL)‐6/JAK/STAT3), fibrosis/extracellular matrix (ECM) remodeling (epithelial–mesenchymal transition (EMT), Collagen‐Containing ECM), and metabolic complications (advanced glycation end‐product/receptor for advanced glycation end‐products (AGE‐RAGE) Signaling, insulin‐like growth factor (IGF) Transport/IGFBPs) (Fig. 4C).

We identified the transcription factor JUN as a central hub, participating in seven of twelve enriched pathways spanning inflammation (TNF‐α/NF‐κB, IL‐6/JAK/STAT3), fibrosis (EMT, AGE‐RAGE), lipid metabolism (PPAR Signaling), and stress response (Hypoxia, Apoptosis) [12] (Fig. 4D). TGFB1 connected inflammatory signaling to extracellular matrix remodeling [13], while IGFBP3 bridged insulin/IGF axis dysregulation to both hypoxia response and fibrotic pathways [33]. The co‐enrichment of PPAR signaling, ethanol metabolic processes, and AGE‐RAGE signaling is consistent with emerging models describing the interaction between metabolic dysfunction and alcohol‐associated liver disease [34]. Key inflammatory mediators (CXCL1, CXCL8, IL18) and cytokine signaling inhibitors (suppressor of cytokine signaling (SOCS)1, SOCS3) indicate possible crosstalk between hepatocytes and immune‐mediated inflammatory signaling networks [35, 36], while collagen genes (COL4A1, COL4A2) and matricellular proteins (VCAN, TNC, THBS1) reflect ongoing fibrogenesis [37, 38]. Notably, MagmaFlow's identification of CXCL1 and CXCL8 as key inflammatory mediators within TNF/NF‐κB signaling was independently corroborated by Guan et al. [39], who demonstrated that hepatocyte‐derived CXCL1 and neutrophil‐expressed CXCL8 drive inexorable inflammation in severe alcohol‐associated hepatitis using the same dataset (Fig. 4D). These shared transcriptional signatures indicate that alcohol‐induced metabolic stress and inflammatory signaling converge on common fibrogenic pathways, contributing to disease progression in ALD (Fig. 4D).

### Multi‐omics cross‐platform integration links validated genes to ALD pathophysiology

MagmaFlow supports visualization of data from multiple omics layers, enabling complementary analysis across molecular platforms. We analyzed ALD datasets spanning global proteomics, phosphoproteomics (MSV000089168), bulk RNA‐seq (GSE142530), and single‐cell RNA‐seq (GSE255772), with each platform processed independently through MagmaFlow's differential expression and pathway enrichment workflows (Fig. 5A–D). Upstream quality control and batch correction were applied to each dataset prior to MagmaFlow input as described in Materials and Methods.

Cross‐platform integration revealed distinct validation patterns: hepatitis vs healthy identified 22 genes across all platforms (Fig. S1A), cirrhosis vs healthy showed 12 genes (Fig. S1B), while hepatitis vs. cirrhosis showed no common genes (Fig. S1C).

Progressive multi‐omics filtering and pathway enrichment analysis revealed disease stage‐specific molecular signatures (Fig. S1D,E). Hepatitis vs healthy filtering (Fig. S1D) progressed through 2462 scRNA‐seq genes, 792 genes after bulk RNA‐seq intersection, 125 genes after proteomics integration, and finally 22 genes validated across all four platforms. These 22 genes mapped to 61 consensus pathways appearing in two or more platforms. TOP 10 ALD‐relevant pathways were selected after excluding nonliver‐specific pathways (Estrogen and ultraviolet (UV) Response) that lacked biological relevance to alcoholic liver disease. Ranked pathways demonstrated metabolic enrichment, including Fatty acid degradation (composite score: 60.4), Metabolism (59.4), and Drug metabolism (55.8), alongside core ECM remodeling pathways such as Collagen‐Containing ECM (70.0) and Epithelial Mesenchymal Transition (70.0). This metabolic pathway signature reflects preserved but dysregulated hepatic metabolic function at early disease stage. Reduced alcohol dehydrogenase (ADH) expression (ADH1A, ADH4) connected to fatty acid and drug metabolism pathways, indicating impaired ethanol metabolism [40], while upregulated cytoskeletal genes (AFAP1, FLNA) associated with focal adhesion pathways suggest active cellular reorganization during early stage injury [41].

Cirrhosis vs healthy filtering (Fig. S1E) progressed from 2716 scRNA‐seq genes through 471, 57, and finally 12 cross‐validated genes mapping to 16 consensus pathways. TOP 10 pathways demonstrated structural damage signatures including hypoxia (51.3), cell‐substrate junction (46.3), actin cytoskeleton (34.1), elastic fiber formation (30.5), and basement membrane (29.8), alongside shared ECM pathways. This structural pathway signature indicates advanced tissue remodeling and scarring at the late disease stage. Sustained upregulation of focal adhesion components (FBN1, FLNA, MYOF) and ECM‐receptor interaction mediators (FBN1, FLNA) was consistent with advanced fibrotic remodeling [41]. Reduced HMOX1 expression may indicate compromised oxidative stress defense mechanisms [42].

Pathway comparison revealed five pathways appearing in both TOP 10 lists: Collagen‐Containing ECM (ranked 1^st^ in both conditions), Epithelial Mesenchymal Transition (2nd in both), Focal Adhesion, Extracellular Matrix Organization, and Endoplasmic Reticulum Lumen, representing conserved core disease mechanisms across ALD progression. The distinct pathway signatures of metabolic dysfunction in hepatitis vs structural collapse in cirrhosis, reflect disease stage‐specific molecular pathophysiology, with hepatitis demonstrating broader pathway activation (61 vs 16 consensus pathways) and greater gene retention (22 vs 12 validated genes), suggesting increasing molecular specificity during disease progression.

The integrated heatmap (Fig. S1F) organized by regulation direction revealed six genes (AFAP1, ANK3, ANXA2, BICC1, MAP1B, MYOF) consistently upregulated across both disease stages and all omics layers. The coordinated regulation of cytoskeletal, membrane trafficking, and stress response genes across molecular layers suggests cellular reorganization as a prominent feature of ALD progression. Notably, phosphoproteomic measurements revealed enhanced fold change magnitudes compared to protein levels, suggesting that phosphorylation can amplify regulatory responses beyond protein abundance changes, though functional validation would be required to confirm these mechanistic relationships.

The hepatocyte scRNA‐seq validation (GSE255772) demonstrated practical utility beyond feature comparison. Independent corroboration by Guan et al. [43] of CXCL1 and CXCL8 as key inflammatory mediators confirms MagmaFlow's capacity to generate biologically meaningful insights aligned with published findings from identical datasets. Multi‐omics pathway integration further revealed stage‐specific molecular signatures, with metabolic enrichment in hepatitis versus structural damage pathways in cirrhosis, alongside five conserved pathways representing core disease mechanisms. These findings demonstrate MagmaFlow's successful incorporation into systems‐level multi‐omics analytical workflows, transforming differential expression data into prioritized gene candidates for experimental validation.

### Comparative feature analysis

**Fig. 6:**
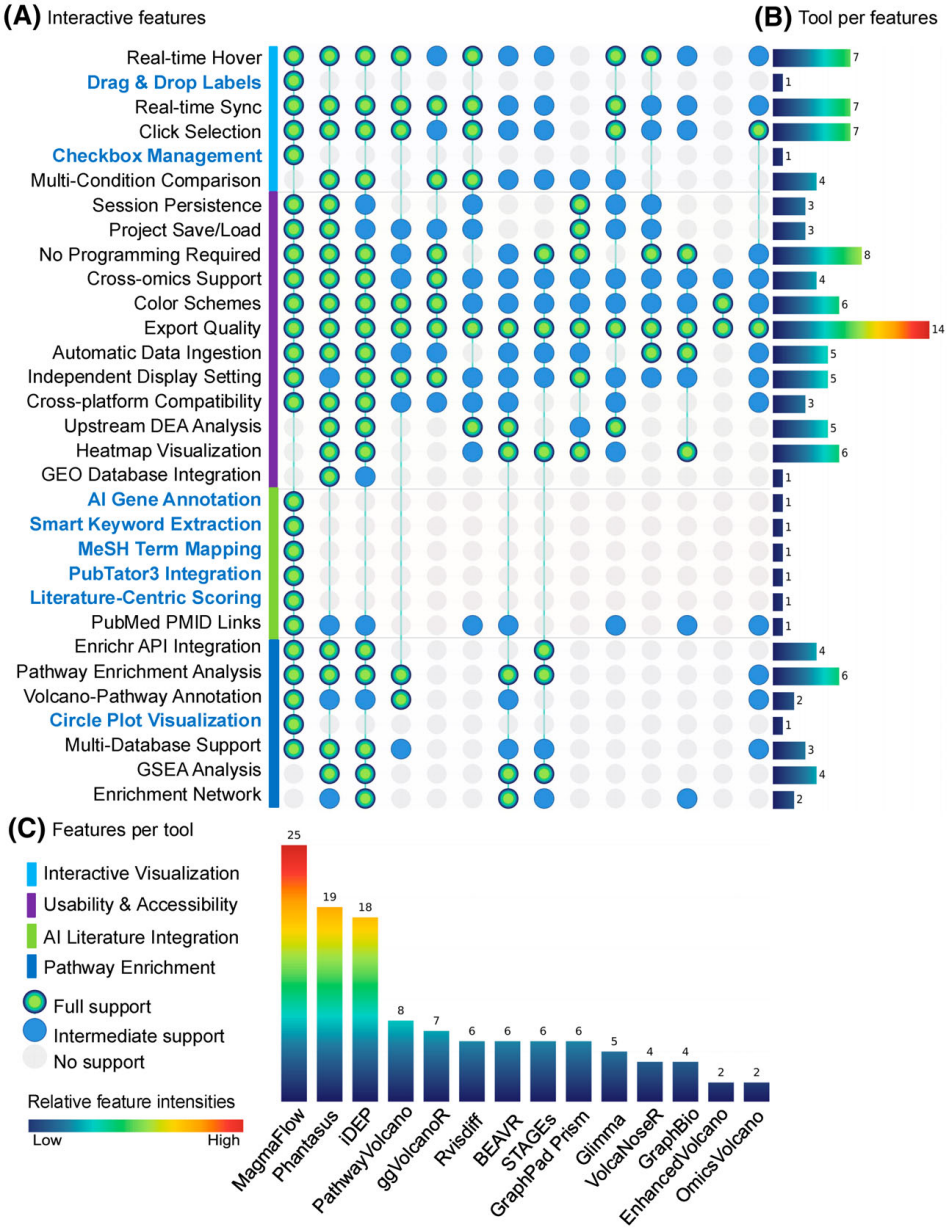
Comparative feature analysis of volcano plot visualization tools. (A) Matrix visualization comparing 31 functional features across 14 volcano plot tools. Features are organized into four categories: Interactive Visualization (6 features in light blue), Usability and Accessibility (12 features in purple), AI‐Powered Literature Integration (6 features in green), and Pathway Enrichment Analysis (7 features in blue). Blue‐green gradient circles indicate full support; blue circles indicate partial support; gray circles indicate absence. Vertical cyan lines connect features within each tool. Nine features are exclusive to MagmaFlow: Drag and Drop Labels, Checkbox Management, AI Gene Annotation, Smart Keyword Extraction, MeSH Term Mapping, PubTator3 Integration, Literature‐Centric Scoring, PubMed PMID Links, and Circle Plot Visualization. Six features present in other tools are not currently implemented in MagmaFlow: Multi‐Condition Comparison, Upstream DEA Analysis, Heatmap Visualization, GEO Database Integration, GSEA Analysis, and Enrichment Network Visualization, and are flagged as active development priorities. (B) Bottom bar chart displays the number of fully supported features per tool (range: 2–25), with MagmaFlow supporting the most features overall (25). (C) Right bar chart shows the number of tools supporting each feature (range: 1–14). Bar charts are color‐coded using the MagmaFlow gradient palette from low (dark blue) to high (red).

We next evaluated MagmaFlow's features compared to available volcano plot visualization tools. A systematic feature comparison across 14 volcano plot visualization tools evaluated 31 features organized into four categories: interactive visualization, usability and accessibility, AI‐powered literature integration, and pathway enrichment analysis. Feature support was classified as full (blue‐green gradient), partial (blue), or absent (gray) (Fig. 6A).

MagmaFlow demonstrated the most comprehensive feature set with 25 supported features, including 9 exclusive capabilities: checkbox‐based gene management, drag‐and‐drop label positioning, smart keyword extraction, MeSH term mapping, PubTator3 integration [14], AI‐powered gene annotation, literature‐centric scoring, PubMed PMID links, and circle plot visualization. This substantially exceeded the next most complete tools: iDEP [44] and Phantasus [45] (18–19 features each), PathwayVolcano [5] (8 features), and other tools (2–7 features each) [8, 9, 10, 46, 47, 48, 49, 50] https://bioconductor.org/packages/EnhancedVolcano (Fig. 6B,C).

Interactive features showed variable implementation across tools: real‐time hover tooltips, synchronization, and click‐based selection were moderately common. Drag‐and‐drop label positioning, which is essential for resolving label overlapping in dense differential expression figures and producing publication‐ready outputs without manual postprocessing, remained exclusive to MagmaFlow, as did the checkbox‐based gene management system that enables real‐time synchronization between gene selection, literature annotation, and pathway enrichment results. AI‐powered literature integration represented the most distinctive differentiator, with all six literature‐related features exclusive to MagmaFlow. While some tools offered pathway enrichment via Enrichr [16, 17, 28] or gene set enrichment analysis (GSEA)‐based analysis, none provided AI‐driven literature contextualization or automated relevance scoring (Fig. 6C).

## Discussion

MagmaFlow addresses a critical gap in differential expression analysis by transforming volcano plots from static endpoints into dynamic, interactive environments where gene annotation, literature evidence, and pathway context converge in a single session. Rather than automating biological interpretation, MagmaFlow is designed to accelerate the hypothesis generation process while keeping the researcher as the central decision‐maker, providing ranked evidence and contextual information that informs experimental prioritization without replacing scientific judgment.

Three architectural innovations distinguish MagmaFlow from current alternatives. First, an integrated gene selection panel functions as a central hub connecting manual gene selection, AI‐powered literature rankings, and pathway enrichment results into a unified annotation layer, enabling a bidirectional analytical workflow where pathway findings guide gene prioritization and the volcano plot contextualizes the results within differential expression statistics. For wet‐laboratory researchers, this means moving directly from thousands of differentially expressed genes to a biologically contextualized shortlist without switching between tools. Second, drag‐and‐drop label repositioning with automatic collision avoidance enables publication‐ready figure generation without manual postprocessing, a capability fundamentally unavailable in R and Python packages that generate fixed, noninteractive plots. Third, AI‐powered literature contextualization via PubTator3 integration remains absent from all 13 compared tools, enabling automated research relevance scoring directly within the visualization environment.

The comparative analysis across 14 tools and 31 features revealed that leading web‐based platforms such as iDEP [44] and Phantasus [45] offer comprehensive upstream analysis pipelines including differential expression analysis from raw counts, heatmap visualization, multi‐condition comparison, and GSEA, but reduce volcano plot visualization to a secondary output without dedicated annotation integration. MagmaFlow occupies a distinct and complementary role focused on the downstream interpretation, ready‐publication dedicated features, and is not designed to replicate these upstream capabilities. Its desktop architecture ensures core functionality remains available offline for sensitive or unpublished datasets, while AI‐powered features require internet connectivity but remain freely accessible without registration or usage limits.

MagmaFlow addresses a specific and practical need in omics data interpretation, providing researchers with an accessible integrated desktop environment for the annotation and functional contextualization of differential expression results. In its current version, the platform processes a single DEA result at a time and does not natively support multi‐omics integration, though its outputs are fully compatible with dedicated multi‐omics workflows. The literature relevance score provides a useful proxy for research prominence within a given disease context, though publication frequency and biological relevance do not always align, and novel or understudied genes may warrant attention beyond their literature score. Pathway enrichment results may be influenced by gene set size and database curation depth, and the circular network visualizations reflect shared gene membership across pathways rather than mechanistically validated crosstalk. These characteristics are common to the broader class of enrichment‐based interpretation tools and reflect the current state of the field.

Planned enhancements for future releases include statistically grounded pathway crosstalk scoring via hypergeometric testing and Search Tool for the Retrieval of Interacting Genes/proteins (STRING) protein–protein interaction (PPI)‐based inter‐pathway interaction analysis [51], support for parallel DEA session imports for multi‐condition comparison, automated enrichment term clustering to reduce ontological redundancy, heatmap visualization, and a cloud‐compatible deployment option to broaden accessibility beyond desktop environments. Long‐term sustainability is supported by KTO‐ULB, which holds the software's intellectual property rights and is actively evaluating licensing and downstream development opportunities. Each stable release is versioned and archived on Zenodo, with the GitHub repository and documentation maintained in synchronization with each release cycle.

## Conflict of interest

The KTO‐ULB holds the intellectual property rights to the MagmaFlow software. The authors declare no conflict of interest.

## Author contributions

CEB study design, software development, research data selection, data analysis, and manuscript writing. AL, EHG, MB, SPS, LB, and AKC data analysis, manuscript reviewing, and editing. ENG study design, data analysis, manuscript reviewing, and editing.

## Supporting information


**Fig. S1.** Multi‐omics Integration and Pathway Enrichment Analysis in Alcoholic Liver Disease.

## Data Availability

The data that support the findings of this study are openly available as detailed below. The datasets reanalyzed in this study were derived from the following resources available in the public domain. MagmaFlow software, documentation, and validation datasets (Zenodo, GitHub): The MagmaFlow software, documentation, and example datasets are openly available in Zenodo at https://doi.org/10.5281/zenodo.20299121 and in the GitHub repository at https://github.com/carlosbuss1/MagmaFlow, where all input datasets, intermediate outputs, and enrichment results used to generate the figures are also deposited. Functional genomics data (GEO, NCBI): Functional genomics data are openly available in NCBI's Gene Expression Omnibus and are accessible through Bulk RNA‐seq https://www.ncbi.nlm.nih.gov/geo/query/acc.cgi?acc=GSE142530 and scRNA‐seq https://www.ncbi.nlm.nih.gov/geo/query/acc.cgi?acc=GSE255772, GEO Series accession numbers GSE142530 and GSE255772. Mass spectrometry proteomics data (MassIVE/ProteomeXchange): The mass spectrometry proteomics data (Global and Phosphoproteomics) were obtained from the ProteomeXchange Consortium (http://proteomecentral.proteomexchange.org) via the MassIVE partner repository, dataset identifier MSV000089168.
