# Supplementary material for: MagmaFlow: A desktop platform for artificial intelligence‐driven expression analysis
Source: FEBS Open Bio. 2026 Jun 23:10.1002/2211-5463.70288. Online ahead of print. doi: 10.1002/2211-5463.70288 (PMC13398583; doi:10.1002/2211-5463.70288)
Supplement: Supplementary file 1 — Fig. S1. Multi‐omics Integration and Pathway Enrichment Analysis in Alcoholic Liver Disease. [file FEB4-9999-0-s001.docx]

**Supplementary Figures.**


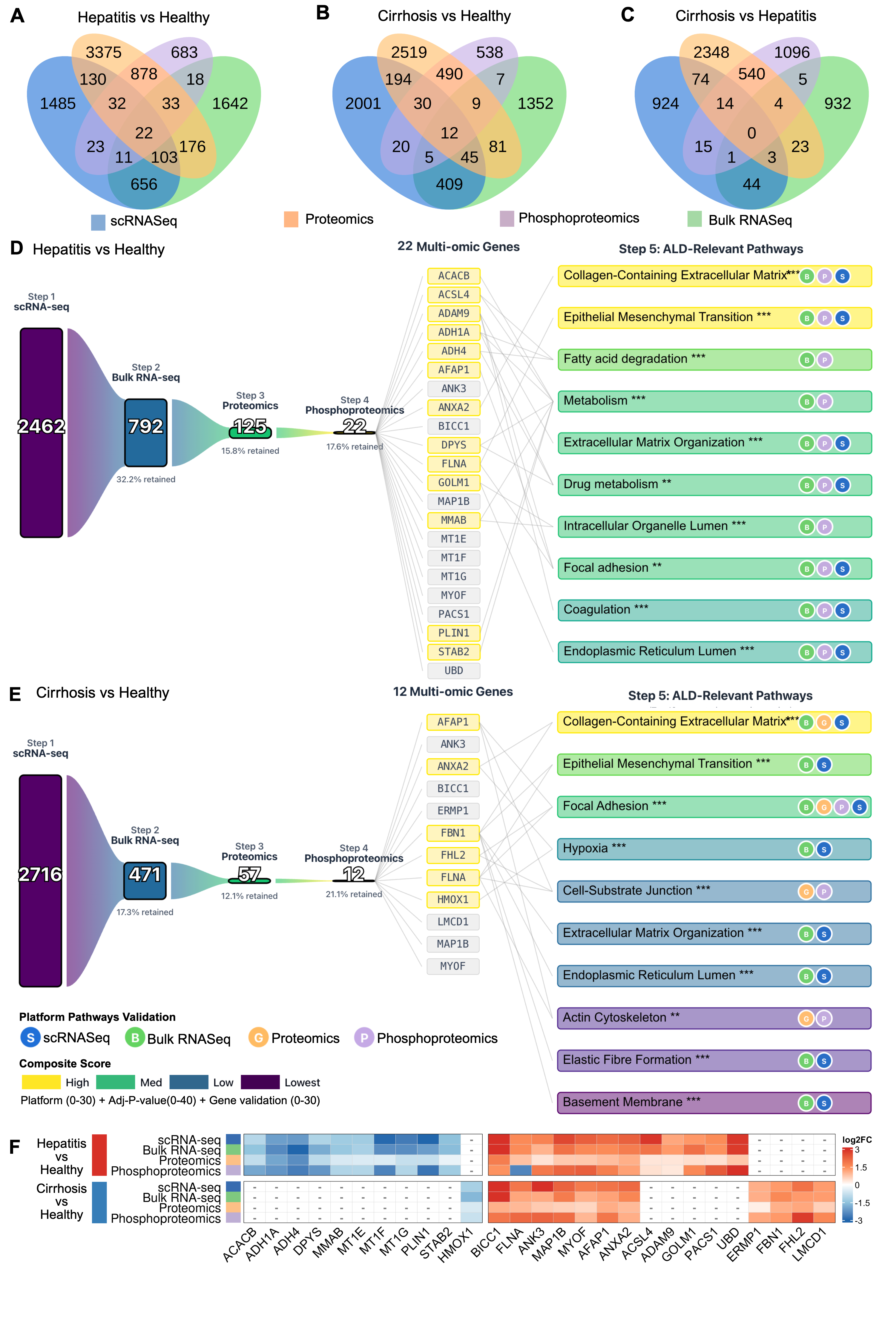


**Figure S1. Multi-omics Integration and Pathway Enrichment Analysis in Alcoholic Liver Disease. (A-C) Multi-omics Gene Overlap. Four-way Venn diagrams showing gene intersections across scRNA-seq, Bulk RNA-seq, Global Proteomics, and Phosphoproteomics (p < 0.05, absolute log2FC > 1). (A)** Hepatitis vs Healthy: 22 shared genes across all four platforms. **(B)** Cirrhosis vs Healthy: 12 validated genes across all four platforms. **(C)** Hepatitis vs Cirrhosis: no overlap due to zero significant genes in Bulk RNA-seq layer. **(D-E) Sankey diagrams visualizing sequential gene intersection through omics layers with mapping to TOP 10 ALD-relevant consensus pathways. Flow progression: scRNA-seq, then scRNA-seq intersect Bulk, then scRNA-seq intersect Bulk intersect Proteome, then All 4 platforms, then TOP 10 pathways. Node heights proportional to gene counts. Gene boxes color-coded: yellow (connected to pathways), gray (no enrichment). Platform badges: Green (Bulk), Blue (scRNA), Orange (Global Proteomics), Purple (Phospho). Pathway bars colored by composite score (viridis scale: yellow = high, purple = low). (D)** Hepatitis vs Healthy: 2,462 then 792 then 125 then 22 genes mapped to 61 consensus pathways (2 or more platforms). TOP 10 show metabolic enrichment: Fatty acid degradation, Metabolism, Drug metabolism, reflecting preserved metabolic function at early disease stage. **(E)** Cirrhosis vs Healthy: 2,716 then 471 then 57 then 12 genes mapped to 16 consensus pathways. TOP 10 demonstrate structural damage: Hypoxia, Cell-Substrate Junction, Actin Cytoskeleton, Elastic Fibre Formation, Basement Membrane, indicating advanced tissue remodeling at late stage. Five pathways shared between conditions (Collagen-Containing ECM, Epithelial Mesenchymal Transition, Focal Adhesion, ECM Organization, ER Lumen) represent conserved disease mechanisms. Pathway significance: three asterisks p < 0.001, two asterisks p < 0.01, one asterisk p < 0.05. Composite scoring: platform validation (0-30 pts) + statistical significance (0-40 pts) + gene multi-platform coverage (0-30 pts). Non-liver-specific pathways excluded. **(F) Cross-platform Validation Heatmap. Log2 fold change heatmap for genes validated across all four platforms. Upper: 22 genes (Hepatitis vs Healthy). Lower: 12 genes (Cirrhosis vs Healthy). Red = upregulation, blue = downregulation, white with dash = missing data. Six genes overlap between conditions (AFAP1, ANK3, ANXA2, BICC1, FLNA, MAP1B, MYOF). Consistent regulation direction across platforms with enhanced phosphoproteomics signal intensity.**
